# ST-V-Net: incorporating shape prior into convolutional neural networks for proximal femur segmentation

**DOI:** 10.1007/s40747-021-00427-5

**Published:** 2021-06-16

**Authors:** Chen Zhao, Joyce H. Keyak, Jinshan Tang, Tadashi S. Kaneko, Sundeep Khosla, Shreyasee Amin, Elizabeth J. Atkinson, Lan-Juan Zhao, Michael J. Serou, Chaoyang Zhang, Hui Shen, Hong-Wen Deng, Weihua Zhou

**Affiliations:** 1 Department of Applied Computing, Michigan Technological University, 1400 Townsend Dr, Houghton, MI 49931 USA; 2 Department of Radiological Sciences, Department of Mechanical and Aerospace Engineering, Department of Biomedical Engineering, and Chao Family Comprehensive Cancer Center, University of California, Irvine, Irvine, CA 92697 USA; 3 Center of Biocomputing and Digital Health, Institute of Computing and Cybersystems, and Health Research Institute, Michigan Technological University, Houghton, MI 49931 USA; 4 Department of Radiological Sciences, University of California, Irvine, Irvine, CA 92697 USA; 5 Division of Endocrinology, Department of Medicine, Mayo Clinic, Rochester, MN USA; 6 Division of Epidemiology, Department of Health Sciences Research, and Division of Rheumatology, Department of Medicine, Mayo Clinic, Rochester, MN USA; 7 Division of Biomedical Statistics and Informatics, Department of Health Sciences Research, Mayo Clinic, Rochester, MN USA; 8 Division of Biomedical Informatics and Genomics, Tulane Center of Biomedical Informatics and Genomics, Deming Department of Medicine, Tulane University, School of Medicine, 1440 Canal Street, Suite 1610, New Orleans, LA 70112 USA; 9 Department of Radiology, Tulane University School of Medicine, New Orleans, LA 70112 USA; 10 School of Computing Sciences and Computer Engineering, University of Southern Mississippi, Hattiesburg, MS 39406 USA

**Keywords:** Quantitative computed tomography, Proximal femur, Segmentation, Deep learning, Convolutional neural networks

## Abstract

We aim to develop a deep-learning-based method for automatic proximal femur segmentation in quantitative computed tomography (QCT) images. We proposed a spatial transformation V-Net (ST-V-Net), which contains a V-Net and a spatial transform network (STN) to extract the proximal femur from QCT images. The STN incorporates a shape prior into the segmentation network as a constraint and guidance for model training, which improves model performance and accelerates model convergence. Meanwhile, a multi-stage training strategy is adopted to fine-tune the weights of the ST-V-Net. We performed experiments using a QCT dataset which included 397 QCT subjects. During the experiments for the entire cohort and then for male and female subjects separately, 90% of the subjects were used in ten-fold stratified cross-validation for training and the rest of the subjects were used to evaluate the performance of models. In the entire cohort, the proposed model achieved a Dice similarity coefficient (DSC) of 0.9888, a sensitivity of 0.9966 and a specificity of 0.9988. Compared with V-Net, the Hausdorff distance was reduced from 9.144 to 5.917 mm, and the average surface distance was reduced from 0.012 to 0.009 mm using the proposed ST-V-Net. Quantitative evaluation demonstrated excellent performance of the proposed ST-V-Net for automatic proximal femur segmentation in QCT images. In addition, the proposed ST-V-Net sheds light on incorporating shape prior to segmentation to further improve the model performance.

## Introduction

Osteoporosis is an initially silent bone disease characterized by fractures of the hip (the proximal femur), spine or wrist. Hip fractures are particularly debilitating and difficult to predict [1]. Quantitative computed tomography (QCT) is used to evaluate osteoporosis and the risk of hip fracture by quantifying bone density and geometry of regions within the proximal femur [2, 3]. QCT is also combined with a structural analysis technique called finite element (FE) modeling to compute the force required to cause fracture of the proximal femur when the bone is subjected to specific loading conditions [4, 5]. Although valuable research has been performed using this methodology, studies of large cohorts are hindered by the need for segmentation of the proximal femur, which typically involves a semi-automated technique requiring time consuming user monitoring and manual intervention. If a fully automated method of segmentation existed, large cohorts of subjects could be analyzed to facilitate new discoveries, such as those involving artificial intelligence.

Proximal femur segmentation has been performed using traditional image processing-based methods. Younes et al. [6] proposed an automatic method for femur segmentation using primitive shape recognition and statistical shape models. They assumed that the femoral head was a sphere and the femoral shaft was a cylinder. However, precision was limited by the primitive shapes and the model only achieved a Dice similarity coefficient (DSC) of 0.87 and an average surface distance (ASD) of 1.480 mm. Xia et al. [7] employed a multi-atlas model for 3D hip joint segmentation on bilateral MR images. The model achieved a DSC of 0.946 and an ASD of 0.846 mm. Arezoomand et al. [8] proposed a semi-automated active model to register the femur volume with a predefined atlas to complete the segmentation, and the model achieved a sensitivity of 0.88. Chandra et al. [9] presented a weighted shape learning approach for deformable models applied to hip joint segmentation, and the model achieved a DSC of 0.98 on MR images. From technical perspectives, most of the traditional methods rely on fine-tuning a large number of hyperparameters and shape priors, and the running time is relatively long [10].

Several deep-learning-based methods have been proposed. Zeng et al. [11] proposed a 3D U-Net with multi-level deep supervision for automatic proximal femur segmentation on 3D MR images of femoroacetabular impingement. Chen et al. [12] used 3D feature-enhanced modules, including edge detection and multi-scale feature fusion modules to automatically extract the proximal femur from CT images. However, existing deep-learning-based methods have not achieved satisfactory results, which motivates us to develop a more precise algorithm to automatically segment the proximal femur. Deep learning models are optimized based on voxel-wise loss; however, smoothness of the segmentation results is not guaranteed. Inspired by the cascade convolution neural network (CNN) proposed by Nanda et al. [13], image segmentation results can be optimized by a multi-step training strategy. In addition, shape is one of the most important geometric attributes for describing objects [14], and incorporating a shape prior restricts the searching space for the model output [15].

In this paper, a deep-learning-based method using a V-Net framework to automatically extract the proximal femur from QCT images was developed and validated. The contributions of the paper are listed below:

1. We proposed a 3D end-to-end V-Net to perform femur segmentation based on QCT images.
2. We integrated the 3D spatial transform network (STN) [16] into the V-Net to restrict the model to generate the segmentation results which were corresponding to the shape priors. The integrated model which contains the V-Net and the STN in a unified network is called as ST-V-Net. By utilizing the shape prior deformation, our model produced plausible segmentation results of the proximal femur.
3. We designed a multi-stage training strategy to train the ST-V-Net which produced the shape priors and refined the segmentation results iteratively. The weights in the V-Net were trained firstly, and then the STN was fine-tuned secondly. After that, the shape priors were generated by applying morphological operations to the V-Net segmentation. Finally, the V-Net and STN were trained jointly while the shape priors were updated simultaneously. The shape priors are not limited by the results generated using dilation operators, and the active shape model [17] and the active appearance [18] approaches are also suitable to generate the shape priors and are easy to be embedded into our ST-V-Net model.

**Fig. 1:**
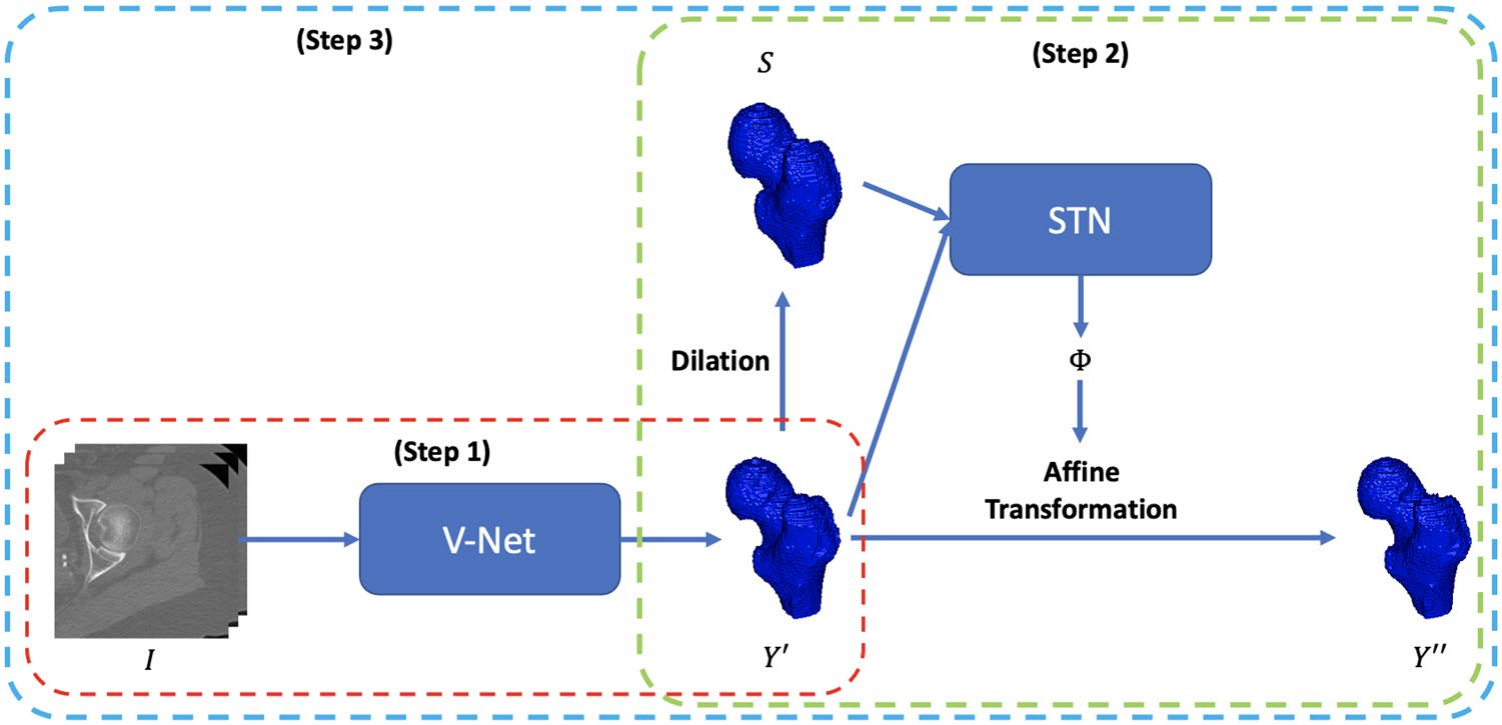
Workflow of the proposed ST-V-Net and corresponding training strategy. ST-V-Net contains a V-Net for image segmentation and a spatial transform network (STN) to restrict the searching space of the segmentation results according to the shape prior $S$. $Y^{\prime}$ is the femur segmented by V-Net from the input $I$ and $Y^{\prime\prime}$ is the femur segmented by ST-V-Net

The workflow of our proposed method for femur segmentation is shown in Fig. 1. We firstly train a V-Net to generate the proximal femur segmentation results and then train a STN to refine the output of the network according to the shape prior, as shown in the red rectangle and green rectangle respectively. After obtaining the optimized weights of both V-Net and STN, we jointly fine-tune the weights of the entire ST-V-Net until it converges, which is depicted in the blue rectangle.

## Methods and materials

### Subjects and data pre-processing

We obtained anonymized QCT scans of the hips of 216 women and 181 men [mean age (range): 60.9 (27–90) years old, and 59.9 (28–90) years old, respectively] from the year 6 follow-up visit of a previous longitudinal study of an age-stratified random sample of Rochester, MN residents [19]. The subjects included here are a subset of that study and were selected for analysis in a previous FE study [20]. Subjects previously provided written informed consent that extended to the analyses presented here. Ninety-five percent of the men and 99% of the women were white.

The QCT scans (Siemens, Sensation 64, 120 kVp; 2-mm-thick slices; pixel size, 0.742–0.977 mm; convolution kernel, B30s; 512 × 512 matrix) were converted to 3-mm-thick slices for consistency and comparison with previous FE studies [4]. Fourier interpolation was performed over groups of three contiguous 2-mm-thick images to obtain six 1 mm-thick contiguous images, followed by decimation (by averaging voxels in successive slices) to create two 3-mm-thick contiguous images.

To obtain the ground truth, the left proximal femur was segmented by one of the authors (TSK) using thresholding combined with an edge following algorithm [21] that was integrated with in-house software to create a user-interactive semi-automated segmentation procedure.

**Fig. 2:**
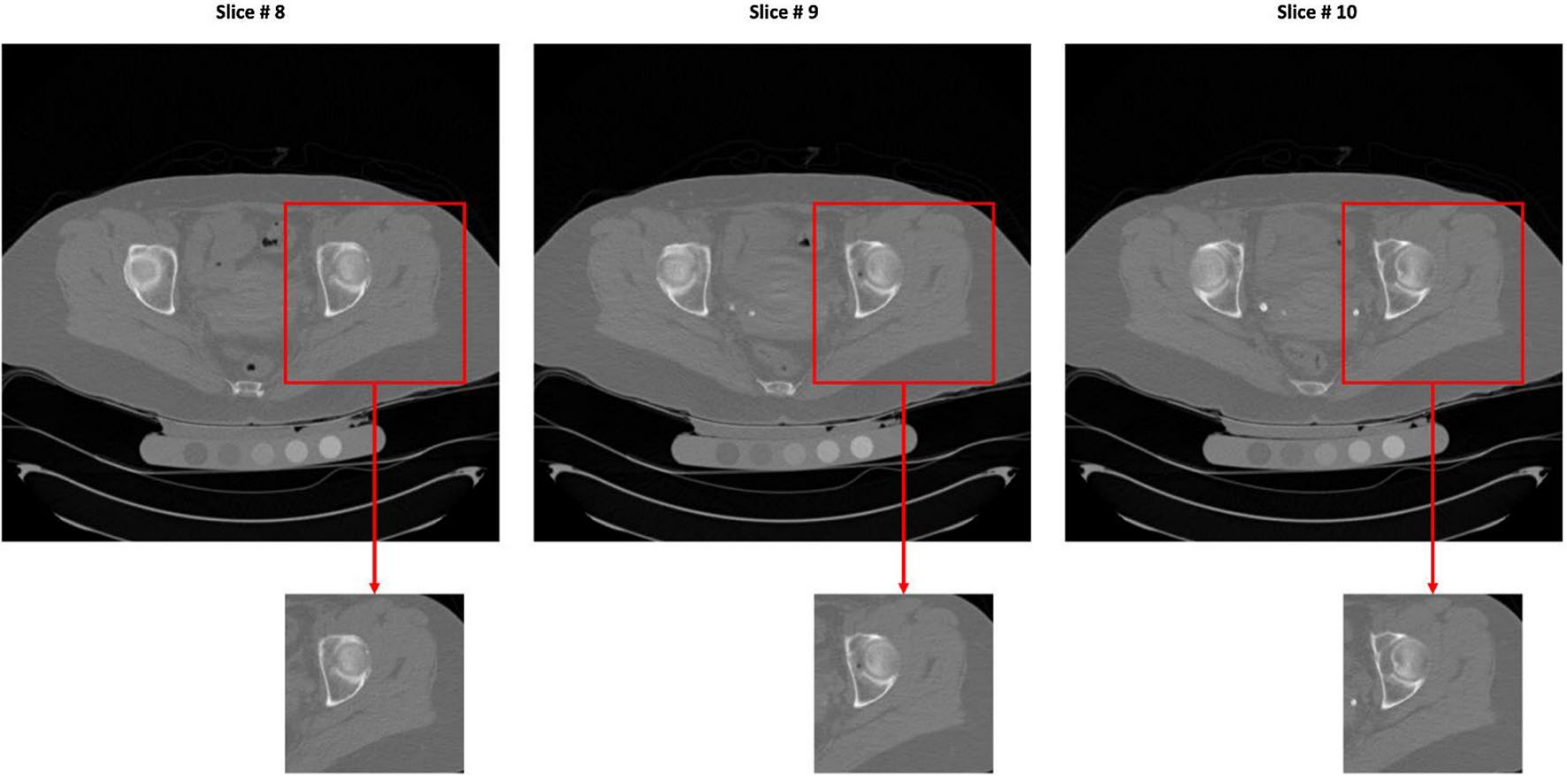
Cropping process for generating the input volume

During processing, the resolution of a single QCT slice is 512 × 512 with 3 mm slice thickness. Only the ground truth of the left proximal femur, which is on the right side of each slice, was annotated, so we manually cropped a rectangular area in the middle-right part of the original image to train our model. The number of slices in the subjects ranged from 37 to 95. The cropping process is shown in Fig. 2.

**Fig. 3:**
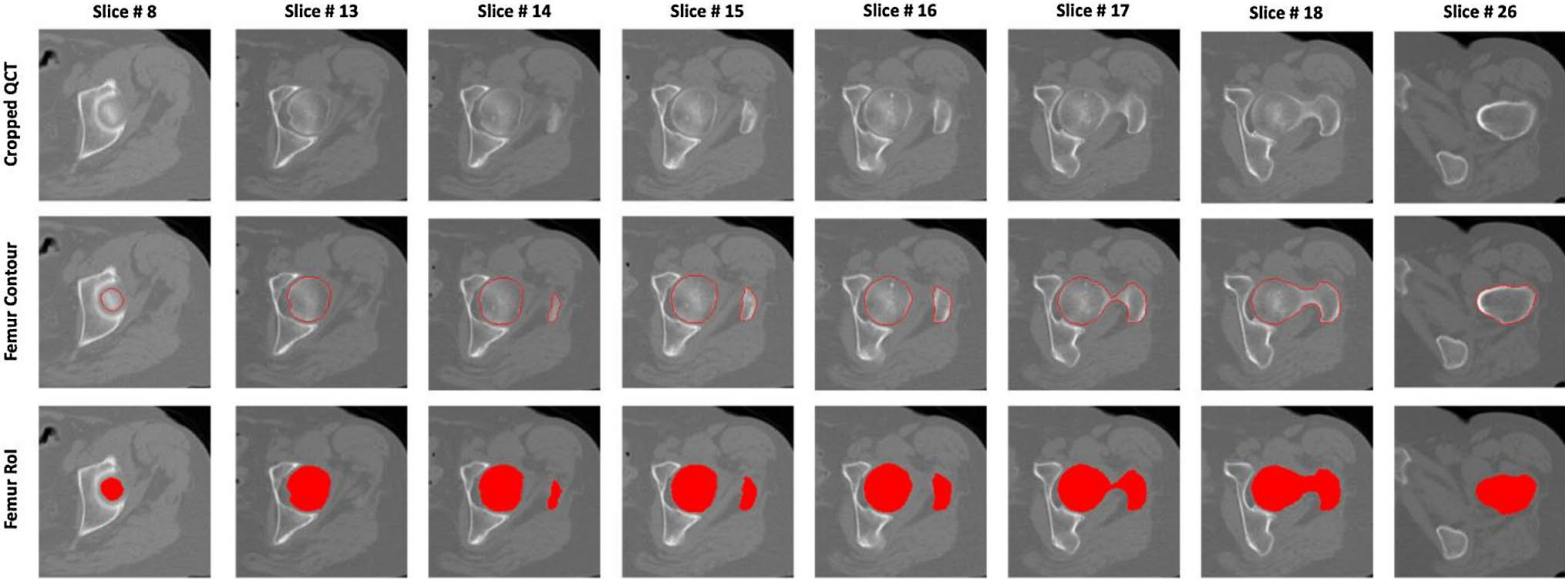
Examples of QCT slices (top row) with corresponding femur contours (middle row) and RoIs (bottom row)

Examples of cropped QCT slices with corresponding contours and the proximal femur region of interest (RoI) are shown in Fig. 3.

### Proposed V-Net model for femur segmentation

**Fig. 4:**
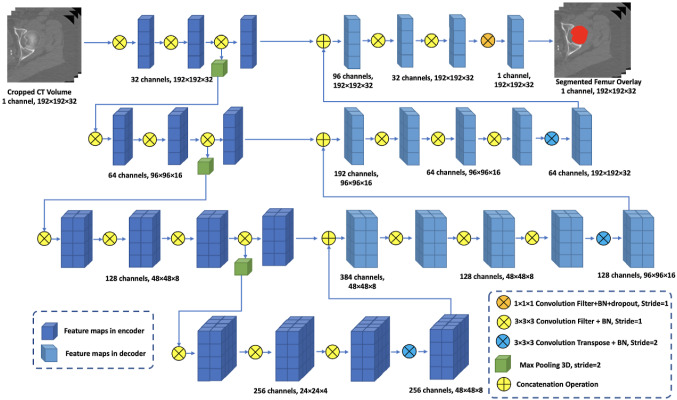
Schematic diagram of the proposed V-Net for femur segmentation. BN represents batch normalization [22]

Our proposed femur segmentation method consists of a volume-based deep learning network—a 3D end-to-end V-Net. The input of the model is a cropped QCT image volume with a size of 192 × 192 × 32. In the model, the 3D V-Net architecture is employed to encode the latent and high-level features of the QCT images and decode the features to generate the final segmentation results. After feeding the cropped volume of a QCT image into the 3D V-Net, a Dice loss, which computes the discrepancy between the generated segmentation result and manually delineated ground truth (gold standard), is used for evaluating the current performance of the model. After that, the gradient of the loss is computed, and back-propagation through the whole flow of the V-Net is employed to fine-tune the model parameters during the training. The workflow of the proposed 3D V-Net is depicted in Fig. 4.

As shown in Fig. 4, the designed V-Net architecture of femur segmentation contains an encoder and a decoder. In detail, the encoder of the V-Net contains three down-sampling layers implemented using max pooling operations, and the symmetric decoder consists of three up-sampling layers implemented by transpose convolution operations. Each feature map after the pooling operation is fed into the decoder through a skip-connection operation, and it is concatenated with the corresponding feature map in the decoder. The number of feature maps in each convolution block is demonstrated in Fig. 4. Before the model output layer is created, a convolutional layer with 1 × 1 × 1 kernel is employed to transfer the feature maps into a probability map with a resolution of 192 × 192 × 32. In addition, before the last convolutional layer is created, a dropout layer is added to prevent the model from overfitting [23]. The output of each voxel of the proposed V-Net is the probability that the particular voxel is contained within the proximal femur. Finally, the OTSU algorithm [24] is employed to convert this probability map into binary segmentation results where ‘1’ represents a voxel in the femur, and ‘0’ indicates the background.

### Spatial transformation model

According to the experimental results (in section Experimental result), even though the designed V-Net achieved a satisfied segmentation result, the model performance was limited by the relatively large slice thickness of the QCT images which reduced the reliability of the segmentation results, especially for the femoral head. According to the difference of the contours between the segmentation results and corresponding ground truth, we found that the segmented femur masks were always smaller than the ground truth, and the surface of the proximal femur was coarse. Our solution is to enlarge the segmentation results and smoothen the surface by applying morphological dilation with a unit diagonal matrix with the size of 5 × 5 × 5. However, without adjusting the volume of the generated masks according to the corresponding proximal femur, the post-processed segmentation results are not accurate. The post-processed segmentation results are denoted as the shape priors for the proximal femur; however, the generated shape priors are required to be deformed according to the ground truth. Hence, a spatial transformation module is designed to predict the transformation parameters so that the shape prior is aligned to the corresponding ground truth, and the output of the network is refined accordingly.

The spatial transformation module computes the non-rigid transformation parameters to align the segmentation results with the ground truth. The aim of the transformation module is to restrict the output of the network according to the shape prior, and a penalty between the deformed output and the ground truth is added into the objective function to optimize the weights for the segmentation model. The input of the transformation module is a two-channel image which contains the segmentation results generated by V-Net and the shape priors. The transformation module computes the non-rigid affine transformation parameters, which are denoted as $\Phi$. STN, which is capable of providing a spatial transformation, is employed as the architecture of the transformation module. STN actively and dynamically transforms an image by generating approximate transformation parameters for each pair of inputs. The spatial transformation module is trained in an end-to-end back propagation manner and is easy to be embedded into the image processing network, such as CNN.

In the proposed ST-V-Net, the input QCT image is defined as $I$, the shape prior is denoted as $S$, and the ground truth is defined as $Y$. $Y^{\prime}$ is the V-Net output and $Y^{\prime\prime}$ is the aligned output. All of them have a size $M \times N \times L$. In our transformation module, the input is the combination of $Y^{\prime}$ and $S$ as a two-channel 3D image with the size of $M \times N \times L \times$ *2*, and the output is the parameter matrix for the affine transformation. Formally, the affine transformation is defined in Eq. 1.

1 $$ \left( {\begin{array}{*{20}c}    {x_{i}^{s} }  \\    {y_{i}^{s} }  \\    {z_{i}^{s} }  \\   \end{array} } \right) = \Phi \left( {\begin{array}{*{20}c}    {\begin{array}{*{20}c}    {x_{i}^{t} }  \\    {y_{i}^{t} }  \\    {z_{i}^{t} }  \\   \end{array} }  \\    1  \\   \end{array} } \right) = \left[ {\begin{array}{*{20}c}    {\begin{array}{*{20}c}    {\begin{array}{*{20}c}    {\Phi _{{11}} }  \\    {\Phi _{{21}} }  \\    {\Phi _{{31}} }  \\   \end{array} } & {\begin{array}{*{20}c}    {\Phi _{{12}} }  \\    {\Phi _{{22}} }  \\    {\Phi _{{32}} }  \\   \end{array} }  \\   \end{array} } & {\begin{array}{*{20}c}    {\begin{array}{*{20}c}    {\Phi _{{13}} }  \\    {\Phi _{{23}} }  \\    {\Phi _{{33}} }  \\   \end{array} } & {\begin{array}{*{20}c}    {\Phi _{{14}} }  \\    {\Phi _{{24}} }  \\    {\Phi _{{34}} }  \\   \end{array} }  \\   \end{array} }  \\   \end{array} } \right]\left( {\begin{array}{*{20}c}    {\begin{array}{*{20}c}    {x_{i}^{t} }  \\    {y_{i}^{t} }  \\    {z_{i}^{t} }  \\   \end{array} }  \\    1  \\   \end{array} } \right) = F_{\Phi } \left( {Y_{i}^{{\prime \prime }} } \right) $$

where $\left( {x_{i}^{s} ,y_{i}^{s} ,z_{i}^{s} } \right)$ are the source coordinates in the input $Y^{\prime}$, $\left( {x_{i}^{t} ,y_{i}^{t} ,z_{i}^{t} } \right)$ are the target coordinates of the output $Y^{\prime\prime}$, $i \in \left[ {1, \ldots ,M \times N \times L} \right]$ is the index of voxel location, and $F_{\Phi }$ is the affine transformation function. The shape transformation module aims at calculating the mapping from source to target and the calculated coordinates are float indexes. A trilinear interpolation is employed to convert the float indexes into integer coordinates, and perform the spatial transformation of the input $Y^{\prime}$ to $Y^{\prime\prime}$. In addition, the trilinear interpolation is used to prevent the re-sampled voxels from being extrapolated to the outside of the 3D volume and guarantee the differentiability of the loss function of the neural network, which is illustrated in Eq. 2.

2 $$ \begin{aligned}   Y_{i}^{{\prime \prime }}  &  = \mathop \sum \limits_{m}^{M} \mathop \sum \limits_{n}^{N} \mathop \sum \limits_{l}^{L} Y_{{mnl}}^{\prime } \;{\text{max}}\left( {0,1 - \left| {x_{i}^{s}  - m} \right|} \right){\text{max}}\left( {0,1 - \left| {y_{i}^{s}  - n} \right|} \right){\text{max}}\left( {0,1 - \left| {z_{i}^{s}  - l} \right|} \right),~ \\     & \;\;\forall i \in \left[ {1, \ldots ,M \times N \times L} \right]~ \\  \end{aligned} $$

and the partial derivatives of the aligned output $Y^{\prime\prime}$ over the input of the 3D STN are:

3 $$ \begin{aligned}   \frac{{\partial Y_{i}^{{\prime \prime }} }}{{\partial Y_{{mnl}}^{\prime } }} &  = \mathop \sum \limits_{m}^{M} \mathop \sum \limits_{n}^{N} \mathop \sum \limits_{l}^{L} {\text{max}}\left( {0,1 - \left| {x_{i}^{s}  - m} \right|} \right){\text{max}}\left( {0,1 - \left| {y_{i}^{s}  - n} \right|} \right){\text{max}}\left( {0,1 - \left| {z_{i}^{s}  - l} \right|} \right) \\    \frac{{\partial Y_{i}^{{''}} }}{{\partial x_{i}^{s} }} &  = \mathop \sum \limits_{m}^{M} \mathop \sum \limits_{n}^{N} \mathop \sum \limits_{l}^{L} Y_{{mnl}}^{\prime } \;{\text{max}}\left( {0,1 - \left| {y_{i}^{s}  - n} \right|} \right){\text{max}}\left( {0,1 - \left| {z_{i}^{s}  - l} \right|} \right)\left\{ {\begin{array}{*{20}l}    {0,} \hfill & {{\text{if}}~\left| {x_{i}^{s}  - m} \right| \ge 1} \hfill  \\    {1,} \hfill & {{\text{if}}~x_{i}^{s}  \le m} \hfill  \\    { - \;1,} \hfill & {{\text{if}}~x_{i}^{s}  > m} \hfill  \\   \end{array} } \right. \\  \end{aligned} $$

and the derivatives of $\frac{{\partial Y_{i}^{{\prime \prime }} }}{{\partial y_{i}^{s} }}$ and $\frac{{\partial Y_{i}^{{\prime \prime }} }}{{\partial z_{i}^{s} }}$ are the same as $\frac{{\partial Y_{i}^{{\prime \prime }} }}{{\partial x_{i}^{s} }}$. Using the trilinear interpolation defined in Eq. (2), the differentiability of the transformation module is guaranteed so that the entire network can be trained by a gradient-based method.

**Fig. 5:**
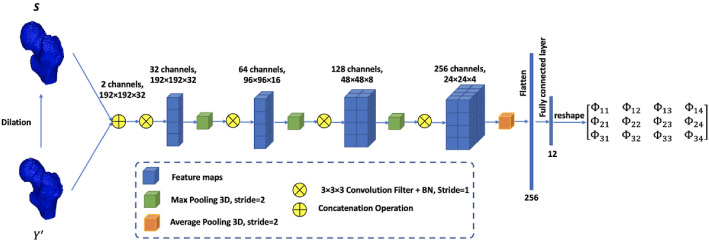
The architecture of the transformation module. *BN* batch normalization

The network architecture of the transformation module is shown in Fig. 5. The input includes the V-Net output and the generated shape prior, and a concatenation layer is employed to combine the two inputs into a two-channel image. The design of the network architecture is inspired by the classification network using CNN developed in [25, 26]. The designed transformation module contains four convolutional layers and four pooling layers to extract features from the concatenated image. Then, a flatten layer is utilized to convert the feature maps into the feature vectors. Finally, a fully connected layer is used to calculate the parameters for the affine transformation.

### Loss function and optimization strategy

The proposed ST-V-Net contains a V-Net for image segmentation and a transformation module for restriction and refinement. Compared with the background of the whole QCT scan, the proximal femur only occupies a small portion of voxels. To address the data imbalance problem, a Dice loss is employed as a part of the objective function [27] for V-Net and the transformation module. A compound loss function, which consists of two Dice losses and an L2 regularization term, is utilized to penalize the difference between the segmentation results and the ground truth, and to prevent the model from overfitting. The Dice loss of the V-Net is used to penalize the discrepancy between the model prediction $Y^{\prime}$ and the ground truth $Y$, while the Dice loss of the transformation module is utilized to penalize the discrepancy between the deformed model prediction $Y^{\prime\prime}$ and the ground truth $Y$.

1. Loss function of the V-Net.

The DSC measures the amount of agreement between two image regions, as defined in Eq. (4),

4 $$ {\text{DSC}} = \frac{{2\left| {G\left( {Y^{\prime}} \right) \cap Y} \right|}}{{\left| {G\left( {Y^{\prime}} \right)} \right| + \left| Y \right|}} $$

where $Y$ is the ground truth, $G$ is the image binarization function, $G\left( {Y^{\prime}} \right)$ is the binary femur segmentation result obtained from the predicted probability map, and the $\left|  \cdot  \right|$ indicates the number of the voxels belonging to the proximal femur. The DSC between the ground truth and segmented result increases as the accuracy of the segmentation increases.

In practice, we employ a gradient descent-based method, which degrades the loss gradually, to optimize the weights of the ST-V-Net. Finally, the Dice loss, defined in Eq. (5), is used as the objective function in pursuit of a higher DSC.

5 $$ L_{{{\text{DSC}}}} \left( {Y^{\prime},~Y} \right) = 1 - \frac{{2\left| {G\left( {Y^{\prime}} \right) \cap Y} \right|}}{{\left| {G\left( {Y^{\prime}} \right)} \right| + \left| Y \right|}} $$

2. Loss function of the transformation module.

The Dice loss, representing the discrepancy between the deformed model prediction $Y^{\prime\prime}$ and the ground truth $Y$, is defined in Eq. (6).

6 $$ L_{{{\text{DSC}}}} \left( {Y^{\prime\prime},~Y} \right) = 1 - \frac{{2\left| {G\left( {Y^{\prime\prime}} \right) \cap Y} \right|}}{{\left| {G\left( {Y^{\prime\prime}} \right)} \right| + \left| Y \right|}} $$

The output of the transformation module is a non-rigid transformation matrix; however, the ground truth is absent. Instead of optimizing the transformation matrix, we first transform the image and compare the difference between the transformed probability maps and the ground truth. Thus, the matrix is optimized.

Our V-Net and transformation module contain 8.6 and 1.4 million weights, respectively. Therefore, to prevent the ST-V-Net from overfitting and accelerate the convergence during training, an L2 regularization term is employed in the objective function [28].

The overall objective function is defined in Eq. (7):

7 $$ L_{{{\text{loss}}}}  = \alpha L_{{{\text{DSC}}}} \left( {Y^{\prime},~Y} \right) + \beta L_{{{\text{DSC}}}} \left( {Y^{\prime\prime},~Y} \right) + \gamma W_{2} $$

where ${\text{W}}$ represents all weights in the ST-V-Net neural network, $\alpha$, $\beta$ and $\gamma$ are the hyperparameters balancing the loss for V-Net, the loss for the transformation module and the L2 regularization term, respectively.

In addition, we design a multi-stage training strategy. In stage I, the weights of the transformation module are fixed, and the optimization is only applied to the V-Net. In stage II, the weights within the V-Net are fixed and the training is applied to the transformation module. The shape priors are generated according to the V-Net output at the beginning of stage *II*. Since the weights of the V-Net are fixed during stage II, the shape priors are fixed, and the generated shape priors can be used during the entire training in stage II. In stage III, the weights of the entire ST-V-Net are trained jointly. During stage III, the shape priors are updated dynamically: if the model achieves a higher DSC, then the shape priors of all training samples are regenerated according to the V-Net output. The designed multi-stage training strategy is depicted in algorithm I.
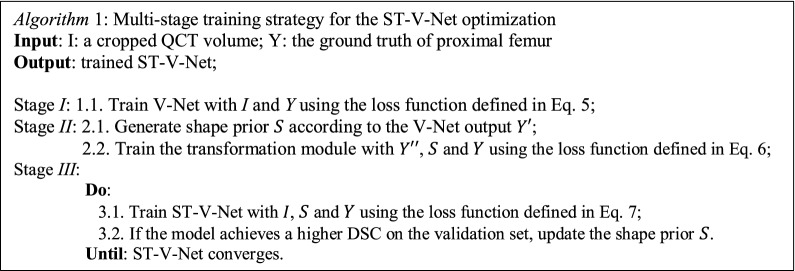


Our ST-V-Net model was implemented in Python using the TensorFlow framework and was trained on a workstation with a NVIDIA Tesla Titan V GPU with 12 GB memory, an I5 CPU and 32 GB RAM. The dropout probability was set as 0.8. The model was trained for 1000 epochs and the weights in the ST-V-Net were optimized using an Adam optimizer [29] with a learning rate of 0.0001.

At the training stage, we separately trained our model on the male cohort, the female cohort and all subjects (All cohort). To overcome overfitting and guarantee the generalizability of the model, we fine-tuned our model with a ten-fold stratified cross-validation on the training and internal validation set. The term ‘stratified’ indicates that male and female subjects were selected according to the proportion of the subjects in each cohort. In each cohort, 10% of the samples were used as the external test set, and the rest data were used as the training set and internal validation set. The parameters of the optimized model were acquired according to the highest DSC on the internal validation set.

Although the dataset contained 397 subjects, the sample size was still insufficient to train a precise 3D segmentation model. To guarantee the robustness of the model, we randomly rotated the cropped volumes by 15 degrees on each axis to augment the data. In addition, during data augmentation, a mirror transform, a brightness transform, a gamma transform, and a Gaussian noise transform were randomly applied to the cropped volumes and the generated samples from each subject were used as the input to our 3D segmentation model for model training [30].

### Evaluation metrics

We evaluated our segmentation model performance using DSC (Eq. 4), sensitivity (SN) and specificity (SP) metrics. Sensitivity measures the proportion of actual positives that are correctly identified as such. Specificity measures the proportion of actual negatives that are correctly identified as such. As the DSC, sensitivity, and specificity approach 1, the segmentation model results increasingly overlap with the ground truth, and the model performance improves.

Since the segmentation of proximal femur from QCT is used for finite element analysis which requires the high accuracy of the femoral surface, we employ Hausdorff distance (HD) and ASD between the segmentation results and the ground truth. HD measures the maximum distance of the model prediction to the nearest point in the ground truth. ASD measures the average of the distances from the points on the surface of the model prediction to the surface of the ground truth. Clearly, a lower HD or ASD indicates the better performance achieved by the segmentation model.

## Experimental results

In our implementation, the inputs to the ST-V-Net are 3D image volumes with a size of 192 × 192 × 32, and each volume contains 32 cropped QCT slices. However, the number of slices varies among the subjects in our dataset. To generate the model prediction of a subject with an arbitrary number of QCT slices, a sliding window scanning technique was adopted which predicted the segmentation from the last axis of the QCT data with an incremental step of 1. That is, if the volume of the QCT image for a specific subject contains 50 QCT slices, the model runs 19 (50–32 + 1) times to generate the final segmentation result. It took 0.09 ± 0.0001 s to predict a volume with the size of 192 × 192 × 32.

**Fig. 6:**
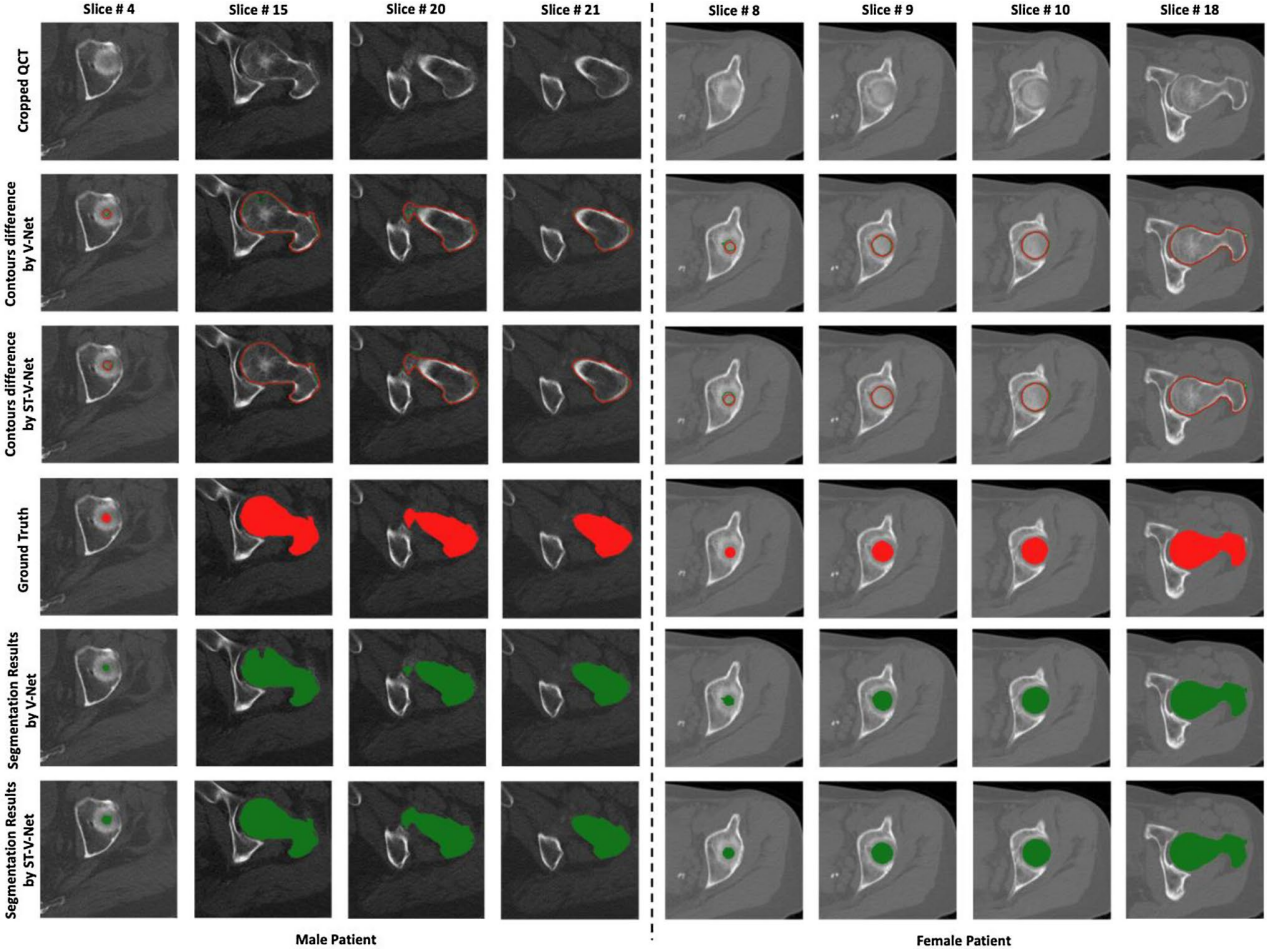
Examples of segmentation results (green) and the corresponding ground truth (red). In the second and third rows, the green contours represent the model predictions corresponding to V-Net and ST-V-Net, respectively, and the red contours show the ground truth

For comparison, the V-Net was used as the baseline model. In our experiments, the hyper parameters in Eq. (7) were set as $\alpha$ = 1, $\beta$ = 0.1 and $\gamma$ = 0.2. Figure 6 juxtaposes the original cropped QCT slices, ground truth and the segmentation results. It can be observed that the contours from our proposed method visually match well with the ground truth.

**Fig. 7:**
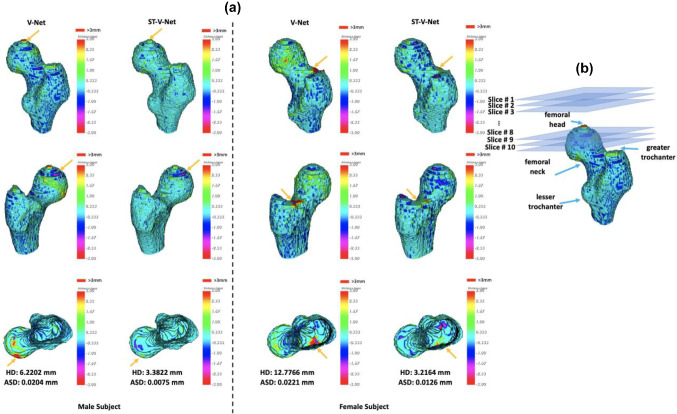
Surface distances for a male subject and a female subject according to the results generated by V-Net and the proposed ST-V-Net. To better visualize the differences, the volumes have been resampled with a new spacing of 1 mm in all axes

Figure 7 visualizes from the three different view angles the distances from the surfaces derived by V-Net and ST-V-Net of two subjects to the ground truth. Corresponding HD and ASD are listed at the bottom of the figure. The range of the surface distance in Fig. 7 is limited to − 3 to 3 mm for visualization.

To measure the model performance, the DSC, HD, ASD, sensitivity and specificity of the segmentation results for the male cohort, female cohort and all subjects are depicted in Table 1.

**Table 1:** Model performance of the proposed ST-V-Net and V-Net baseline

Model	Cohort	DSC	SN	SP	HD (mm)	ASD (mm)
V-Net	All	0.9815 ± 0.0009	0.9906 ± 0.0033	0.9990 ± 0.0000	9.144 ± 2.096	0.012 ± 0.006
	Male	0.9863 ± 0.0012	0.9865 ± 0.0011	0.9991 ± 0.0001	18.748 ± 3.181	0.013 ± 0.005
	Female	0.9847 ± 0.0009	0.9827 ± 0.0023	0.9993 ± 0.0001	12.403 ± 1.667	0.015 ± 0.025
ST-V-Net	All	0.9888 ± 0.0047	0.9966 ± 0.0013	0.9988 ± 0.0001	5.917 ± 1.412	0.009 ± 0.004
	Male	0.9911 ± 0.0033	0.9947 ± 0.0016	0.9991 ± 0.0001	9.626 ± 2.064	0.008 ± 0.003
	Female	0.9890 ± 0.0047	0.9951 ± 0.0019	0.9991 ± 0.0001	3.897 ± 2.526	0.009 ± 0.004

## Discussion

### Performance analysis

As shown in Table 1, our ST-V-Net generated excellent results. Compared to V-Net, the proposed ST-V-Net significantly reduced the surface distance errors (HD and ASD), and improved the voxel overlaps (DSC and SN). For all subjects and for the male cohort, the HD was reduced from 9.144 to 5.917 mm, and from 18.748 to 9.626 mm, respectively. For the female cohort, there was also a reduction in HD (3.897 mm by ST-V-Net vs. 12.403 mm by V-Net).

According to the surface distances rendered by pseudo-colors in Fig. 7a, the distance errors in the femoral head, femoral neck, lesser trochanter and lesser trochanter were significantly reduced. From Fig. 6, the contour differences in the slices were smaller in the ST-V-Net than those in the V-Net. Especially for slice # 15 of the male subject in Fig. 6, the ST-V-Net generated a smoother and more precise contour of the proximal femur than the V-Net did.

Table 2 further compares our methods with existing approaches. The statistical shape model [6] and active shape model [7] only achieved an ASD of 1.480 mm and 0.846 mm, respectively. The method based on Bayes decision rule [30] achieved a DSC of 0.9155 and an ASD of 1.220 mm. All of these traditional image processing-based methods were inferior to the deep learning-based approaches in Table 2. In [12], a multi-scale fully convolutional neural (FCN) was employed to capture the global features and generate the segmentation results in narrow joints. In [31], a U-Net-based network and a Softmax classifier were used to classify femoral voxels into the target and background. Even without the spatial transform module, our V-Net was superior to existing deep learning-based methods. Compared with other approaches that we investigated, we had a large-scale dataset, and our proposed model achieved a higher DSC, a lower HD and a lower ASD.

**Table 2:** Segmentation performance compared with existing approaches

Category	Authors	Method	Data	Sex	Slice thickness (mm)	DSC	HD (mm)	ASD (mm)
Traditional image processing	Younes [6]	Statistical shape model	20 CT	–	–	0.8700	10.530	1.480
	Xia [7]	Active shape model	38 MR	–	0.7	0.9460	–	0.846
	Cheng [32]	Bayes decision rule	110 CT	M&F	1.5	0.9155	–	1.220
Deep learning	Chen [12]	3D CNN	150 CT	M&F	1.32–1.85	0.9688	–	0.410
	Deniz [31]	3D CNN	36 MR	–	1.5	0.9500	7.880	0.390
	V-Net (baseline)	3D CNN	397 QCT	M&F	3	0.9815	9.144	0.012
	Present study	3D CNN	397 QCT	M&F	3	0.9888	5.917	0.009

*DSC* dice similarly coefficient, *HD* Hausdorff distance, *ASD* average surface distance

All of these findings demonstrated that by incorporating shape priors and using affine transformation to refine the network output, the segmentation results in ST-V-Net were successfully aligned to the ground truth so that the results were more precise. In addition, it can be observed that the model performance was better in the male cohort than in the female cohort. This occurred because, in our dataset, the female subjects had a lower bone density and thus lower image contrast, making the segmentation task more difficult.

### Shape prior for the refinement of femur segmentation

The transformation module successfully refined the output of the network according to the shape prior; accordingly, the penalty between the deformed output and the ground truth was important to optimize the weights for the ST-V-Net. STN in our proposed ST-V-Net is effective for optimizing segmentation results.

In addition, a reliable shape prior is important for the ST-V-Net. Since the loss between the aligned shape prior and the ground truth contributes a lot to the total loss, a precise shape prior is required. It should be noted as well that the shape prior is highly correlated to the specific segmentation task. We chose to use the shape prior generated by a dilation operator because the contours generated by the V-Net were smaller than the ground truth and the dilation operator can enlarge and smoothen the mask. To make the proposed ST-V-Net more generalizable, the shape priors should be generated according to original images rather than the V-Net output. In the near future, we will investigate other methods to generate the shape prior, such as the active contour model [8].

A trilinear interpolation was used in the shape transformation module. It could be replaced by other interpolation methods. A commonly used method is the B-Spline interpolation [33], but this method relies on a large number of parameters [34]. In contrast, trilinear interpolation contains limited parameters and has already achieved satisfactory performance. Thus, we adopted trilinear interpolation in our ST-V-Net.

### Clinical application

QCT imaging is one of the most powerful methods for assessing bone quality in the proximal femur. With rapid and accurate three-dimensional (3D) segmentation of the proximal femur using deep learning, features such as bone mineral density (BMD) and geometry, measurement of cortical and trabecular bone, and finite element analysis (FEA)-computed bone strength (fracture load), have the potential to be integrated into risk stratification for clinical decision-making [4].

The results of this study are promising. With further development to achieve reliable results at the head of the proximal femur, studies of large cohorts of subjects could be analyzed to provide additional insights into evaluating hip fracture risk, the effectiveness of medication and other factors.

### Limitation

In our dataset, the slice spacing was much greater than the pixel size so the context information from neighboring slices was not well utilized, reducing the performance of the V-Net model. As shown in Fig. 7b, where the CT slices are indicated by slice 1, slice 2, slice 3 … sequentially, the size of the femoral head within neighboring slices changes dramatically because the slope of the CT scan slices is close to the slope of the femoral head surface. Because of the large slice thickness and the dramatic change in the size of the femoral head between slice 8 and slice 9, the image slices could not precisely reflect the shape variation. As a result, most errors occurred where the slope of the bone surface was close to the slope of the CT scan slices and where contrast was low due to low bone density such as in the femoral head. To further improve the performance of our segmentation model, the most direct approach is to reduce the slice thickness.

In addition, the shape prior generation can be improved with more sophisticated methods [8]. The shape prior transformation module presented in our approach is an affine transformation. A deformable transformation, such as the B-Spline transformation [33], could be applied to boost the performance of our ST-V-Net.

## Conclusion and future work

We proposed a ST-V-Net to automatically and precisely segment the proximal femur in a QCT scan. The proposed model achieved a DSC of 0.9888, a sensitivity of 0.9966 and a specificity of 0.9988. For the surface distance, the ST-V-Net achieved an HD of 5.917 mm and an ASD of 0.009 mm. Therefore, this method has the potential for clinical use.

In the future, we will further improve the performance of our model to generate more precise segmentation results, especially for the areas with large shape variations. As an option, we will try other algorithms to generate the shape priors and use them to improve our proposed ST-V-Net. Our method will also be extended to other medical image segmentation tasks.
